# Genomic analysis of a 1 Mb region near the telomere of Hessian fly chromosome X2 and avirulence gene *vH13*

**DOI:** 10.1186/1471-2164-7-7

**Published:** 2006-01-16

**Authors:** Neil F Lobo, Susanta K Behura, Rajat Aggarwal, Ming-Shun Chen, Frank H Collins, Jeff J Stuart

**Affiliations:** 1 Indiana Center for Insect Genomics, University of Notre Dame, Notre Dame, Indiana, 46556, USA, and Purdue University, West Lafayette, Indiana 47907, USA; 2 Department of Biological Sciences, Galvin Life Sciences Building, University of Notre Dame, Notre Dame, Indiana 46556, USA; 3 Department of Entomology, Purdue University, West Lafayette, Indiana 47907, USA; 4 Department of Entomology, 505 S Goodwin Ave., University of Illinois, Urbana-Champaign, Il 61801, USA

## Abstract

**Background:**

To have an insight into the *Mayetiola destructor *(Hessian fly) genome, we performed an *in silico *comparative genomic analysis utilizing genetic mapping, genomic sequence and EST sequence data along with data available from public databases.

**Results:**

Chromosome walking and FISH were utilized to identify a contig of 50 BAC clones near the telomere of the short arm of Hessian fly chromosome X2 and near the avirulence gene *vH13*. These clones enabled us to correlate physical and genetic distance in this region of the Hessian fly genome. Sequence data from these BAC ends encompassing a 760 kb region, and a fully sequenced and assembled 42.6 kb BAC clone, was utilized to perform a comparative genomic study. *In silico *gene prediction combined with BLAST analyses was used to determine putative orthology to the sequenced dipteran genomes of the fruit fly, *Drosophila melanogaster*, and the malaria mosquito, *Anopheles gambiae*, and to infer evolutionary relationships.

**Conclusion:**

This initial effort enables us to advance our understanding of the structure, composition and evolution of the genome of this important agricultural pest and is an invaluable tool for a whole genome sequencing effort.

## Background

The Hessian fly (*Mayetiola destructor*) is an important insect pest of wheat (*Triticum *spp.). As a member of the gall midge family (Cecidomyiidae) it belongs to the dipteran suborder Nematocera, which also includes mosquitoes, midges, black flies and fungus gnats. Widespread outbreaks of the Hessian fly occur at irregular intervals in many parts of the world [1]. In the United States local outbreaks cause extensive losses nearly every year. The status of the Hessian fly as an agricultural pest, its behavior and its evolutionary relationship to other insects make it an excellent candidate for genome sequencing.

The complexity of the Hessian fly genome is manifested by the presence of two distinct classes of chromosome: E chromosomes and S chromosomes [2]. The E chromosomes vary from 32 to 45 in number, and are germ line limited. The composition of these chromosomes is still unknown. It has been hypothesized that they function to provide additional copies of genes required for oocyte and embryonic development [3]. It has also been suggested that they are largely composed of parasitic DNA adapted to the Hessian fly's post-zygotic mechanism of establishing X chromosome number, as described below [4]. The S chromosomes compose the more conventional portion of the Hessian fly genome and are present in both the germ line and the soma. They consist of two autosomes (A1 and A2) and two X chromosomes (X1 and X2). The S chromosomes contain the genes that are necessary for the housekeeping and specialized functions associated with each somatic cell type, including the avirulence (*Avr*) genes and other genes that are important in the insect's interactions with wheat. A haploid complement of S chromosomes consists of approximately 160 Mb of DNA [5]. The X chromosomes compose approximately 46% of the S genome. A preliminary understanding of the composition and structure of the S genome would be imperative for a whole genome sequencing effort.

Chromosome imprinting and chromosome elimination are both involved in the anomalous behavior of the Hessian fly genome [4,6]. All Hessian fly zygotes begin life with a diploid set of S chromosomes and a complement of E chromosomes. Chromosome imprinting is evident as the E chromosomes and the paternally derived S chromosomes are eliminated from the primary spermatocytes during spermatogenesis. There is no genetic recombination in males. Thus, every spermatoctye contains only the maternally derived set of S chromosomes. Chromosome imprinting is also evident when the male and female somatic karyotypes are established. During the fifth nuclear division of the embryo, the E chromosomes are eliminated from all presumptive somatic nuclei [7]. A male somatic karyotype (A1 A2 X1 X2/A1 A2 O O) is established if the paternally derived X1 and X2 chromosomes are eliminated from the presumptive somatic nuclei along with the E chromosomes. A female somatic karyotype (A1 A2 X1 X2/A1 A2 X1 X2) is established if the paternally derived X chromosomes are maintained in the presumptive somatic nuclei when the E chromosomes are eliminated. X-chromosome elimination is controlled by maternal genotype. Thus, most female Hessian flies produce families that are either all female or all male.

Wheat breeders and wheat geneticists have worked for decades to discover and incorporate cultivar-specific Hessian fly resistance genes into wheat in an effort to manage this pest [8]. Unfortunately, their many successes have been limited by the evolution of Hessian fly genotypes that are unaffected by those resistance genes. By investigating the genetics of this problem [9] and by virtue of its similarity to the genetics of certain obligate bacterial and fungal plant pathogens [10-12], the following working hypothesis has emerged [13]: Hessian fly and wheat have a gene-for-gene relationship whereby loss-of-function mutations in certain Hessian fly genes (broadly called avirulence or *Avr *genes) enable the flies to overcome the resistance conferred by specific alleles of a corresponding set of genes (broadly called resistance or *R *genes) in wheat. For example, mutations in the *Avr *gene *vH13 *permit the survival of larvae feeding on wheat genotypes carrying the *R *allele *H13*. Hessian fly larvae lacking those mutations die as they attempt to feed on the same wheat genotypes. At least 31 *R *genes have been discovered in wheat [14]. *Avr *genes corresponding to 5 of these *R *genes have been genetically mapped in the Hessian fly genome [5,15]. Although neither *R *genes in wheat nor *Avr *genes in the Hessian fly have been cloned, genetic analysis suggests that the wheat genes for Hessian fly resistance encode receptors that interact, alone or in concert with other factors, with the products of the Hessian fly *Avr *genes. This interaction elicits a biochemical cascade that results in plant resistance and the death of Hessian fly larvae attempting to feed on the plant. Recessive mutations in the *Avr *genes appear to enable the insect to avoid detection by the plant. This leads to larval survival and plant damage on plants that would otherwise be resistant to Hessian fly attack. The evolutionary and functional characterization of important Hessian fly genes, such as *Avr *genes, would include an understanding of their functional and evolutionary relationships to homologues in sequenced genomes.

We discovered markers sufficiently close to *vH13 *to attempt chromosome walking for the purpose of cloning, for the first time, an *Avr *gene from an insect [15]. Two bacterial artificial chromosome (BAC) libraries were constructed and walking began in both directions from the most tightly linked DNA marker (22–124). Though the libraries lacked clones containing segments of the genome between *vH13 *and 22–124, we generated a contig of approximately 1 Mb in the opposite direction. Our objectives in the present study were to better understand the molecular structure and composition of this genome using the sequence information garnered. We correlate physical and genetic distance in this region of the Hessian fly genome, and evaluate this segment of DNA for structural and genetic similarities to the sequenced dipteran genomes of the fruit fly, *D. melanogaster*, and the malaria mosquito, *A. gambiae*. This serves as an effective platform towards a whole genome sequencing effort as well as enables an understanding of the functional and evolutionary relationships between these 3 Dipterans.

## Results

### Chromosome walk

**Figure 1:**
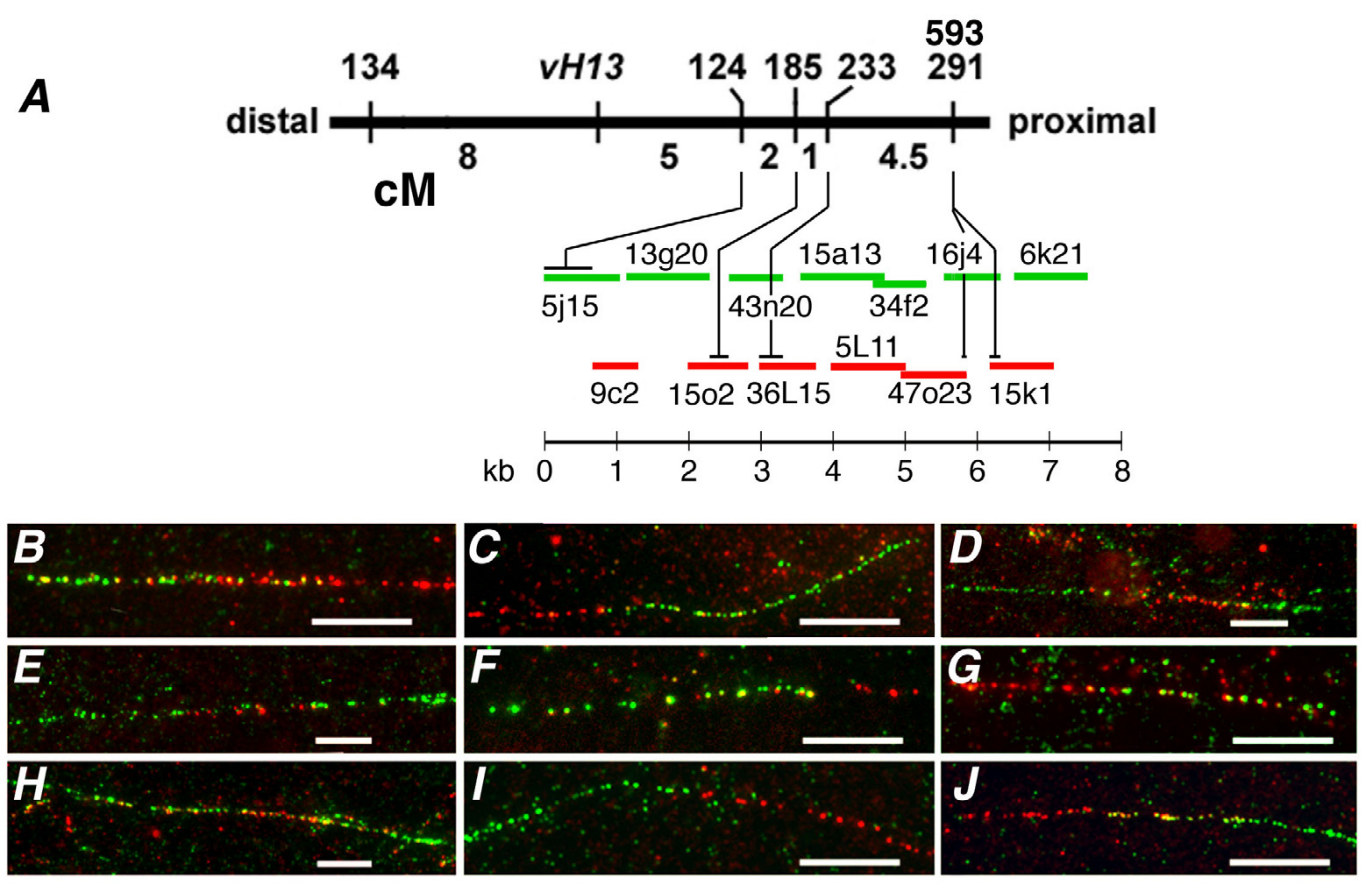
Contig 124-right chromosome walk. (A) Chromosome walking was initiated at STS marker 124 and proceeded 13 steps to the right in reference to the Hessian fly genetic map near avirulence gene *vH13 *shown as a thick black horizontal line. Vertical lines indicate the genetic positions of Avr gene *vH13 *and five previously mapped STS markers (134, 124, 185, 233, and 291; Rider et al. 2002) along the genetic map. A sixth marker (593), developed from the end sequence of Mde47o23-SP6, co-segregated with STS 291 in the same mapping population. Units shown below the genetic map are Kosambi centiMorgans (cM). Horizontal green and red lines shown below the genetic map represent BAC clones in the 124-right contig. The line colors correspond to the color the clones fluoresced in Fiber-FISH experiments (B-J). The scale (in kb) shown below these lines indicates the approximate length of each of these clones in the contig. The length of each clone and its overlap with flanking clones was determined by these experiments. Horizontal black lines shown above the BAC clones in the contig indicate the approximate positions of the STS markers in the contig. (B-J) The fluorescence observed at the intersection of adjacent BAC clones (shown in A) on single DNA fibers. The length of the white bars is 10 μm. B) clones Mde5j15 (green) and Hf9c2 (red), C) clones Hf9c2 (red) and Hf13g20 (green), D) clones Hf13g20 (green), Mde15o2 (red), and Mde43n20 (green), E) Mde43n20 (green), Mde36L15 (red), and Hf15a13 (green), F) clones Hf15a13 (green), and Hf5L11 (red), G) clones Hf5L11 (red), and Mde34f2 (green), H) clones Mde34f2 (green), Mde47o23 (red), and Hf16j4 (green), I) Hf16j4 (green), and Hf15k1 (red), and J) Hf15k1 (red), and Hf6k21 (green).

**Figure 2:**
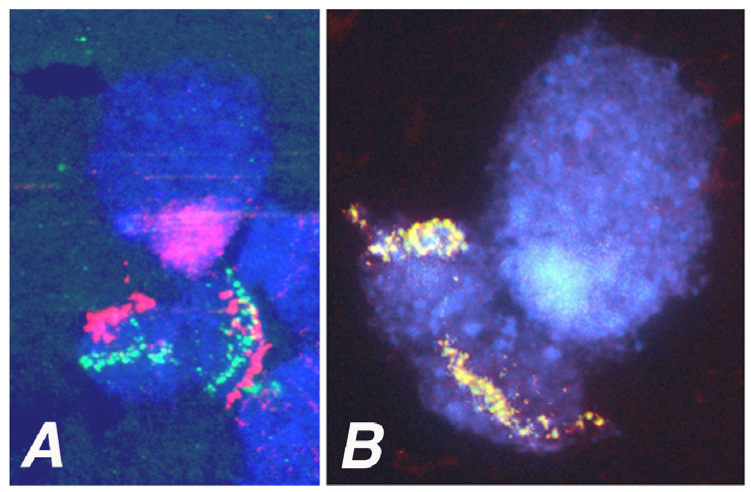
In situ hybridization of BAC clones to the short arm of Hessian fly polytene chromosome X2. (A) BAC clone Mde44o9 (STS 233) green fluorescence and BAC clone 37L3 (STS 134) red fluorescence. (B) Overlapping BAC clones Hf15k1 and Hf15k2 (STS 291); overlapping green and red fluorescence appears yellow.

Chromosome walking was initiated by screening the BAC libraries with STS marker EAC/MCAC-124 (hereafter referred to as 22–124, Fig. 1). Three clones were identified in the *Mde *library and none in the *Hf *library. A chromosome walk then proceeded in one direction while walking in the opposite direction was prevented by an absence of clones in both libraries (Fig. 1). In the direction in which walking was possible, 13 steps were taken, identifying an average of 3.8 ± 2 clones per step and a total of 50 BAC clones. FISH was performed after each step to confirm that the clones were located on the short arm of chromosome X2 (Fig. 2). To determine the orientation and relative lengths of overlapping BAC clones in the walk, a PCR-based method was used. This method utilized oligonucleotides designed after the sequences of BAC-end fragments as primers (described below) and BAC clone DNA as template. Fiber FISH experiments were performed to determine if the walk resulted in a single contig of Hessian fly genomic DNA (Fig. 1). BAC clones representing each step in the walk were used as probes in these experiments. They conclusively demonstrated that a single contig was identified. They were further used to estimate the lengths of the 13 clones and their overlap in the contig (Table 1). The Fiber-FISH determined BAC lengths were slightly greater (1.9%) than those made by CHEF gel electrophoresis (data not shown). The entire contig had an estimated length of 760 kb.

The orientation of the contig with respect to the genetic map was determined using STS markers developed and positioned on the genetic map in a previous investigation [15], and a new marker developed from the DNA sequence at the SP6 end of BAC clone *Mde47o23*. The BAC clones containing each of these markers were identified in separate PCR experiments that used each BAC clone as template and with the primers of each marker (data not shown). The relative positions of the STS markers in the contig were determined with these data (Fig. 1). This analysis clearly showed that the contig extended in the direction away from *vH13*, and that the relative positions of the STS markers on the genetic map corresponded with their relative physical positions in the contig. The lengths of the BAC clones that contained each STS marker and the overlap of those clones with adjacent clones in the contig were used to determine the limits of the STS marker positions (in kb) in the contig (Fig. 1). These limits were then used to determine the minimal and maximal distances between each pair of STS markers in the contig (Table 2). These values were then used to estimate the physical distance between each pair of markers. These values generally fell below 10 cM/Mb, but ranged from 7.6 to an unusually high 22 cM/Mb.

### BAC clone *Mde8i18*

**Figure 3:**
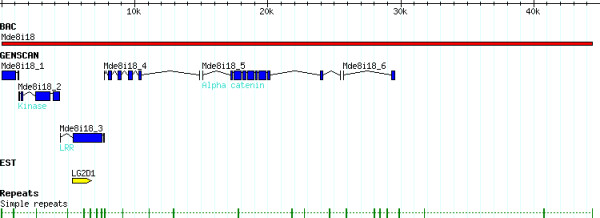
Annotation of *Mde8i18*. The *Mde8i18 *BAC clone was assembled into a 42.6 kb sequence (red bar). All predicted transcripts (blue) are named numerically (*Mde8i18_1-6*). The EST LG2D1 (yellow) corresponded to Mde8i18_3. Repeats identified in this sequence are identified by green bars.

To obtain sequence for a genomic analysis of the contig, a relatively small BAC clone within the contig was selected for sequencing. Sequences generated from the selected clone, *Mde8i18*, clone were assembled into a 42,642 bp contig [GenBank: DQ208194] (Fig. 3). This contig has a minimum of 5× coverage (in both directions) and > 20× sequencing coverage on average. *Mde8i18 *had a G+C content of 31.5%. The assembled sequence had 24 simple repeats (di and tri and tetra-nucleotide). No transposon sequences were found in this genomic segment.

Six putative coding regions (*Mde8i18.1-6*, see Additional file 1) were found following exon prediction (Genscan 1.0) and database searches. A similarity-based search of a Hessian Fly Expressed Sequence Tag (EST) database identified an EST (LG2D1) that corresponded to predicted peptide *Mde8i18_3*. There were 6 predicted transcripts (*Mde8i18_1-6*, see Additional file 1) in this sequence (Fig. 3). Predicted transcripts were compared to nucleotide and protein databases for putative functional assignment.

**Figure 4:**
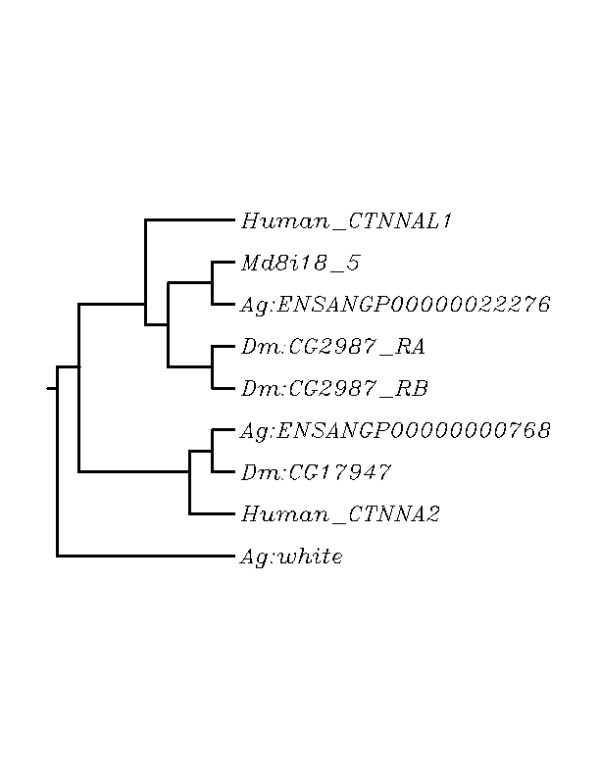
Phylogenetic tree analysis of *Mde8i18-5 *orthologs. The dendrogram was constructed using the sequences that fit the criteria for ortholog determination. There were 2 members of this gene family in the *Anopheles *and *Drosophila *genome (ENSANGP00000022276 and ENSANGP00000000768, and CG2987 and CG17947 respectively). CG2987 had 2 associated transcripts (RA and RB). Human orthologs of the proteins (CTNNAL1 and CTNNAL2) were included to verify orthology predictions. The *A. gambiae white *gene is the outgroup. Md8i18_5 was most similar to the predicted *Anopheles *ortholog, and then to the *Drosophila *ortholog followed by the human ortholog. The second member of this gene family grouped separately verifying that the most likely *Anopheles *and *Drosophila *genes.

Putative peptides *Mde8i18_1 *and *Mde8i18_6 *were partial predictions and were at either end of the BAC clone (Fig. 3), and did not demonstrate similarity to proteins in the database. Predictions *Mde8i18_4 *and *Mde8i18_6 *had no identifiable protein domains. Three other predictions had significant hits to proteins in the *Anopheles *and *Drosophila *genomes. The *Anopheles *and *Drosophila *sequences with the greatest similarity to *Mde8i18_2 *and *Mde8i8_5 *were orthologs of each other [16]. *Mde8i18_2 *(Md_GCN2) was similar to the *Drosophila *GCN2 gene (dGCN2, CG1609) and to its *Anopheles *ortholog – XM_320188. *Mde8i18_5 *belongs to the Vinculin family of proteins and its putative ortholog was the *A. gambiae *alpha-catenin-related gene (XP_309552) and the *D. melanogaster *CG2987 gene. This Hessian fly sequence had two hits in both the *Drosophila *and *Anopheles *genome with putative orthologs having a significantly better E value. The *Anopheles *and *Drosophila *genes were orthologs of each other while the second hits in each genome were orthologs as well. To verify the results, the phylogenetic analysis included the Human orthologs of these genes as well as the *Anopheles white *gene as an outgroup (Figure 4, see Additional file 2). *Mde8i18_3 *had Leucine-rich repeats (LRR) and possessed significant similarity to the *Toll *family of proteins. This sequence was almost identical to a Hessian fly EST, LG2D1 (MSC).

### BAC clone end sequences (BES)

To obtain additional sequence with which to make further comparisons, the end sequences of 40 BAC clones in the contig were determined. This effort resulted in 62 high quality BAC end sequences (BES) [GenBank: DU135285-DU135346] with an average length of 647 bp after vector removal and end-trimming. Sequencing failures were attributed to low BAC DNA yield. Some BESs were found to overlap with each other and the fully sequenced Mde8i18 sequence. These overlaps served to anchor the ends of these BACs. All other BESs represent 41,241 bp of non-overlapping unique sequence contained within the contig. This value is ~81 Kb when *Mde8i18 *BAC sequence is included representing approximately 10% of the entire contig. These sequences have an overall G+C% of 32%, approximately the same as that seen for the Mde8i18 sequence.

Hessian fly putative ortholog determination was based on similarity of protein sequence. Orthologs are genes in different species that have evolved from a common ancestral gene by speciation and usually retain the same function. Identification of orthologs is critical for reliable prediction of gene function in newly sequenced genomes. To minimize the level of false positives, putative orthologs had to meet a strict set of requirements (see Methods, Table 3). Eleven BES had significant similarity (BLASTX and TBLASTN) to proteins in the *Anopheles *and *Drosophila *genomes (Table 3, Fig. 4). With the exception of *Mde8i18_4 *(LRR protein) and *Mde8i18_3*, all TBLASTN searches recovered only 1 significant hit in each genome. There were 5 sequences that had similarity to genes that belonged to single gene families in both the *Anopheles *and *Drosophila *genomes (Table 3). These Hessian fly sequences included BESs *Mde29L21_SP6*, *Mde5j15_Sp6*, *Mde1502_T7*, *Mde33n3_T7 *and *Hf15a13_T7*. Six BESs belonged to multi-gene families in either or both of the other Dipteran genomes. Putative orthologs could not be postulated when there was more than one gene family member in both the *Anopheles *and *Drosophila *genomes. However, in some cases, like BES *Hf3a11_T7*, the *Drosophila *ortholog (*gprs*, CG18471) belonged to a single gene-family while there were 2 genes in the family in *Anopheles*. A putative ortholog was postulated as only one of these *Anopheles *genes (XM_320284) was already determined by Ensembl to be the ortholog of the *Drosophila *gene. In addition, the Hessian fly sequence had significantly high similarity (BLASTX and TBLASTN) to only this *Anopheles *gene. Putative orthologs were also determined for BESs *Hf10f11_T7*, *Hf4F24_SP6*, *Mde29L21_T7 *and *Mde36j2_SP6 *(Table 3). *Mde36j2_T7 *overlapped with the LRR family protein, *Mde8i18_3*.

## Discussion

An *in silico *comparative genomic analysis was performed utilizing Hessian fly genetic mapping, genomic sequence and EST sequence data along with data available from public databases. We assembled a 760 kb region on the short arm of chromosome X2 and related physical distance to genetic distance. We sequenced, assembled and analyzed a 42.6 kb BAC clone (*Mde8i18*) from the Hessian fly (Fig. 1). This sequence data was supplemented with 62 BESs, contained within the contig and encompassing the *Mde8i18 *BAC clone, to perform a comparative genomic study. Exon prediction combined with BLASTX and TBLASTN analyses revealed significant similarities to the *A. gambiae *and *D. melanogaster *genomes (Fig 2). Mosquito and fly putative orthologs were determined for 6 of 11 sequences that demonstrated similarity (Table 1). The higher similarity of the Hessian fly sequences (based on BLAST values) to the *Anopheles *putative orthologs indicated that the Hessian fly is closer related to *A. gambiae *than *D. melanogaster*.

High-resolution physical mapping of DNA by in situ hybridization (Fiber-FISH) is a well established method of physical genome mapping that has been used with mammals, yeast, cloned fragments, and plants [18-23]. The ratio of genetic to physical distance (determined by this method), indicates an unusually high recombination rate (~10 cM/Mb) in this region. Though the recombination rate is not constant across a particular genome, it averages at about 1.5 cM/Mb in both *Drosophila *and humans [24]. Recombination rates that are unusually high are seen in insects like the honeybee, which demonstrate genome wide recombination rates as high as 19 cM/Mb [25]. To confirm the Fiber-FISH determined physical distances and hence the high recombination rate, 7 BAC clones that were measured by Fiber FISH were also measured by CHEF gel electrophoresis. The total distance of 7 clones in the contig as measured by CHEF gel electrophoresis was 591 kb (data not shown) whereas their total distance measured by F-FISH was 603 kb. Fiber-FISH measurements were slightly greater (1.9%) than estimates made by CHEF gel electrophoresis confirming the initially measured recombination rate. This higher recombination rate may be due to its telomeric location where recombination is usually higher [24] or to the specific nature of this part of this genome.

The assembly of the Hessian Fly *Mde8i18 *BAC clone was accomplished using highly stringent parameters [26,27] as this was the first genomic sequence assembly effort in this insect. The presence of a low frequency of randomly dispersed sequence mate-pairs with inconsistencies in either size or orientation was attributed to random error during library generation or assembly. These sequences were discarded, as their omission had no effect on the assembly. The stringent measures taken ensure the accuracy of this assembly and support all ensuing predictions and conclusions.

The G+C content of 32% observed here is comparable with 35.2% seen in *A. gambiae *[28] as well as that seen in *D. melanogaster *(41.1%) [29]. The 6 predicted transcripts in this region represent a gene density of 1 gene in ~7.5 kb indicating the presence of a higher number of genes in this region of the *Mayetiola *genome than that of the Dipterans, *A. gambiae *(1/11 kb) (F.H.C.) and *D. melanogaster *(1/13 kb) [29] in general. The observation that the Hessian fly genome has a higher gene density, lower transposon content (none observed) and a small genome size (156.5 Mb/haploid genome [30] fits in with the linear relationship of genome size and transposon content [31]. The smaller size and repeat content of this genome will facilitate the more efficient assembly of this genome in a genome sequencing effort.

Three of the 6 predicted peptides on this BAC clone had no similarity to proteins in the databases. This may reflect a portion of the transcriptome that is unique to the Hessian fly, or genes that have either diverged significantly or have been lost from other genomes being presently studied. In addition, the complete annotation of the 2 partial predictions may reveal sequence with similarities to known proteins. The predicted peptide *Mde8i18_3 *was found to have significant similarity to an EST sequence – LG2D1. Though this predicted peptide had significantly similar to the *Toll *family of proteins, the *Toll*-related EST sequence differed slightly from the prediction. This result validates the importance of EST projects [24,32,33] in not just supporting but also the improvement of *ab initio *gene prediction.

To compare transcriptomes and infer phylogeny, we BLASTed Hessian fly sequences to the genomes of *A. gambiae *and *D. melanogaster*. Sequences with significant similarity were evaluated for possible orthologous relationships. It is important to note that all orthology inferred here is putative as the complete genome of the Hessian fly has not been sequenced. Sequences have been postulated as orthologs only after meeting stringent criteria [17]. We have combined BLASTX and TBLASTN searches with phylogenetic analyses and linked these results to the additional feature of using orthologous relationships between the *A. gambiae *and *D. melanogaster *genomes to determine putative Hessian fly orthologous sequences (See Figure 4 and Additional file 2 for an example of phylogenetic analysis). The strict criteria used here leads us to believe that we have minimized false positives. Hessian fly sequences exhibited varying levels of similarity to both genome. *Mde8i18_5 *was virtually identical to its *Anopheles *ortholog (E-0.0) (Figure 4). The extent of similarity to the *Anopheles *sequence points to this protein having a conserved and important role in the two insects. At the lower limits of detection were the *Hf4f24_SP6 *sequence and its similarity to the odorant binding proteins of *Anopheles *(OBP14) and *Drosophila *(Pbprp2). The relative low similarity seen here is likely due to pheromone binding proteins being highly individual and specific for different insect species. In addition the expansion of odorant protein families in various insects leads us to conclude that an orthologous relationship is tentative at best and can only be confirmed with an entire genome annotation. Differing levels of similarity between orthologs amongst genomes would be due to varying evolutionary selective pressures on individual sequences in specific genomes.

Based on amino acid and phylogenetic analyses (Figure 4), Hessian fly sequences were found to demonstrate a higher similarity to *Anopheles *sequences than to *Drosophila *sequences. This supports the argument that the lineage that gave rise to the Nematocera (lower Dipterans including *Anopheles *and *Mayetiola*) diverged after the Brachycera (higher Dipterans including *Drosophila*) split off the ancestral Dipteran lineage [34].

BESs have been used to build detailed comparative physical maps with mammals [35,36]. A preliminary look at the sequences with high similarity demonstrate that there were spread across the *Drosophila *and *Anopheles *genomes (data not shown). Though 17% (11/64) of Hessian fly BESs demonstrated significant amino acid similarity to the *A. gambiae *and *D. melanogaster *genomes, realistic syntenic relationships cannot be inferred in the absence of an entire Hessian fly genome. Previous studies have looked at synteny seen between Dipterans [17,41,42], Anophelines [43] and Drosophilids [44]. The lack of synteny as compared to that seen in mammalian studies [35,38,39] suggests that even though insect genomes may contain highly similar transcripts, evolutionary divergence may correspond to recombination resulting in break fusion events and the resulting translocation of chromosomal arm segments followed by extensive paracentric inversions within the chromosome. The only genes that would retain linear order would be those that were either tightly linked or if their proximity was essential to their function.

## Conclusion

This represents the most extensive analysis of the Hessian fly genome to date, illustrating the importance of comparative genomic analyses to understand evolutionary and genetic relationships. It also provides us with an understanding of the architecture of this genome thereby serving as a platform for a whole genome sequencing effort. This study focused on a ~1 Mb genomic region on the X2 chromosome of the Hessian fly. The relationship between physical and genetic distance revealed an unusually high recombination rate which may be due to its location at the telomeric end of the chromosome or may be a phenomenon specific to this particular region. Hessian fly transcripts identified possessed significant similarity to those in the *A. gambiae *and *D. melanogaster *genomes. Putative orthology was triangulated among all three genomes, inferring evolutionary relationships. The higher similarity seen between *Anopheles *and *Mayetiola *transcripts supports their closer evolutionary relationship and suggests that the higher Dipteran split occurred prior to the divergence of the Hessian fly and mosquito. The variable amount of similarity and seen between putative orthologs comments on evolutionary pressures exerted. The low transposon content as well as a structure not unlike sequenced genomes demonstrates that a WGS effort for this small genome is feasible. Such an effort would enable further evolutionary and comparative sturdies and would allow the characterization of Hessian fly genes such as *vH13 *thereby having a economic and agricultural implications.

## Methods

### Chromosome walking

A chromosome walk near the telomere of the short arm of Hessian fly chromosome X2 was initiated using STS marker EAC/MCAC-124 [15] as a probe to screen 2 Hessian fly BAC libraries (Mde and Hf) [5,45,46]. BAC clone Mde5j15 (Fig. 1) was one of three clones identified by this library screen. The SP6-end of this clone was used as probe in the first step of the chromosome walk that resulted in the contig reported here.

### Hessian fly BAC library screening

The isolation of DNA probes from the ends of BAC clones for BAC library screens are described below. Approximately 25 ng of each fragment (100 to 800 bp long) were gel purified (QIAEX II Gel Purification Kit (QIAGEN)) and labeled in separate random priming reactions with 32-P [dATP] (Random Primer Labeling Kit (Strategene)) according to the manufacturer's recommendations. Nylon filter arrays of the BAC libraries were prepared at the Purdue Genomics Center with a Qpix robot (Genetix). Nylon filters were prepared for hybridization by incubation for 4 hours at 60°C in 25 ml of hybridization solution (0.1 M Sodium Phosphate, 20 mM Sodium Pyrophosphate, 5 mM EDTA, 0.1% SDS, 10% Sodium Dextran Sulfate, 1 mM o-phenanthroline, 500 μg/ml Heparin Sulfate, 50 μg/ml denatured salmon sperm DNA, and 50 μg/ml yeast RNA) in a hybridization oven. Denatured probe was added to the same solution and incubated with the filters at 60°C for 16 hours. After hybridization, the membranes were washed (0.5% SSC solution with 0.1% SDS) and exposed to Cyclone Storage Phosphor Screens (Packard) for 2 hours in the dark at room temperature. Digital images of the hybridizations were produced (Packard Cyclone Storage Phosphor System).

### Isolation of BAC-end fragments for chromosome walking

A modification of AFLP-PCR [47] was used to preferentially amplify sequences from the ends of the inserts of in the BAC clones. To prepare DNA template for these reactions, individual BAC clones were first restriction digested to completion with *Eco*RI and *Mse*I. The resulting fragments were then ligated to either an *Eco*RI linker or an *Mse*I linker in separate reactions. The sequences of the double stranded *Eco*RI, (5'-CTCGTAGACTGCGTACC-3'; 3'-CATCTGACGCATGGTTAA-5') and *Mse*I (5'-GACGATGGAGTCCTGAG-3'; 3'-TACCTCAGGACTCATT-5') linkers were identical to those developed for AFLP-PCR. Each DNA template was then used in four separate PCRs that utilized different combinations of primers: 1) a primer complementary to the *Eco*RI linker (GACTGCGTACCAATTC) and a primer complementary to the SP6 site (TATTTAGGTGACACTATAG) in the BAC vector (pBeloBAC); 2) the same *Eco*RI primer and a primer complementary to the T7 site (TAATACGACTCACTATAGGG) in the BAC vector, 3) a primer complementary to the *Mse*I linker (GATGAGTCCTGAGTAA) and the SP6 primer, and 4) the *Mse*I primer and the T7 primer. The amplification of *Mse*I-*Mse*I and *Eco*RI-*Eco*RI fragments were less efficient than the amplification of SP6-*Eco*RI, SP6-*Mse*I, T7-*Eco*RI, and T7-*Mse*I fragments. Therefore, most reactions resulted in the presence of a single visible amplicon corresponding to BAC-end fragments with either an SP6 or T7 site at one end and an *Eco*RI or *Mse*I site at the other end. These were gel purified and used as probes in library screens as described above.

### Fluorescence *in situ *hybridization (FISH)

Polytene chromosomes were isolated from the salivary glands of second instar Hessian fly larvae and slides prepared [48]. Probes were prepared by labeling BAC clone DNA (~1 μg) with either biotin- or digoxigenin-conjugate dUTP (Roche) by nick translation. Hybridizations were performed with 40–100 ng of denatured probe DNA in 10 μl of hybridization solution (10% dextran solution, 2× SSC, 40% formaldehyde, and 20 ug of Herring sperm DNA) at 37°C for 12 to 15 h. Detection was performed using Alexa Fluor (Molecular Probes) conjugated anti-biotin and rhodamin conjugated anti-digoxigenin. Digital images were taken using UV optics on an ORCA-ER (Hammamatsu) digital camera mounted on an Olympus BX51 microscope, and MetaMorph (Universal Imaging Corp.) imaging software.

### Fiber-FISH

To prepare nuclei for Fiber-FISH, 2 ml of 2^nd ^instar larvae were ground to a fine powder in liquid nitrogen with a pre-cooled mortar and pestle. The powder was mixed with 10 ml chilled Nuclei Isolation Buffer (NIB, 10 mM Tris-HCl pH9.5, 10 mM EDTA, 100 mM KCl, 0.5 M sucrose, 4 mM spermidine, 1 mM spermine, 0.1% mercapto-ethanol) and then the solution was passed through a series of progressively smaller nylon meshes (beginning with a 250-μm mesh and proceeding through a 149-μm, a 49-μm, and finally a 20-μm mesh; Small Parts Inc., Miami Lakes, Florida) in a chilled funnel. NIB (1 ml) containing 10% (v/v) Triton X-100 was then gently mixed into the filtrate and centrifuged at 2,000 × g for 10 m at 4°C. The nuclei pellet was suspended in 10 ml NIB and filtered through 49- and 30-μm nylon meshes. The filtrate was gently mixed with 1 ml NIB containing 10% Triton X-100, and the solution centrifuged at 2,000 × g for 10 m. at 4°C. The supernatant was decanted and the pellet resuspended in 1 to 5 ml of a solution containing 1:1 NIB:glycerol.

To extend target DNA fibers over a glass microscope slide, 1 to 5 μl of prepared nuclei suspension was placed in 80 μl NIB. The nuclei were gently mixed into the solution and then centrifuged at 3,000 × g for 5 m. The supernatant was removed and the pellet was suspended in 2.5 μl of phosphate buffer (PBS, 10 mM sodium phosphate, pH 7.0; 140 mM NaCl). The suspension was then placed across one end of a clean poly-L-lysine glass microscope slide (Sigma-Aldrich) and allowed to air dry until the solution appeared sticky (5 to 10 m). 8 μl of STE lysis buffer (0.5% SDS, 5 mM EDTA, 100 mM Tris, pH 7.0) was placed on top of the nuclear suspension and incubated for 4 m at room temperature. The solution was then slowly dragged down the surface of the slide with the edge of a clean coverslip that was held just above the slide's surface. This preparation was then air dried for 10 m at room temperature, fixed in fresh 3:1 100% ethanol: glacial acetic acid for 2 min, and baked at 60°C for 30 m.

The DNA probe was prepared using nick translation, and denatured in hybridization solution as described for FISH. Probe in hybridization solution (10 μl) was applied to each slide, covered with a 22 × 22 mm coverslip and sealed with rubber cement. After the cement had dried, the slides were placed on a heated surface at 80°C for 3 m. They were placed in a pre-warmed humid chamber in an oven for 2 min at 80°C and then overnight at 37°C.

Detection of biotin-labeled probes was performed using three layers of antibodies to amplify the green signal: 1) AF488-streptavidin, 2) biotin anti-streptavidin, and 3) AF488-streptavidin. Detection of digoxigenin labeled probes was performed with two layers of antibodies: 1) mouse anti-digoxigenin, and 2) AF568 anti-mouse. Fluorescence microscopy and imaging were performed as described for FISH.

### Sequencing and analysis of the Mde8i18 BAC clone

The strategy used by Lobo et al., 2003, was employed to sequence the *Mde8i18 *BAC clone (DQ208194). Two random libraries were constructed by partially digesting the BAC clone with *Sau3A1 *or *Tsp509 I*, and cloning 2–5 kb fragments into pLitmus28i (NEB). Two 9–12 kb partial libraries were similarly constructed. A directional library, constructed by cloning all completely digested EcoR1 fragments, served as a scaffold to assemble the BAC sequence. Direct BAC sequencing was used to anchor the ends of the sequence. Plasmids cloned from all libraries were sequenced from both ends of the inserts with standard M13 forward and reverse primers using ABI Big Dye Terminator v3, and reactions were analyzed on the ABI Prism^® ^3700 DNA Analyzer. Sequencing data was evaluated, trimmed and assembled using SEQMAN II software package (DNASTAR Inc.) [49]. Gaps were filled by primer walking. The assembled sequence was analyzed for repetitive elements and transposon sequences using Repeatmasker and CENSOR [50] and was annotated with both *ab initio *gene prediction and algorithms based on sequence similarity. GENESCAN 1.0 [51], GENEID 1.1 [52]and FGENES 1.0 [53] were used with default parameters and the human training dataset. To avoid over-prediction, genes were only accepted if they were predicted by at least two algorithms or, if they were predicted by one algorithm and were also similar to known ESTs, cDNAs, or proteins. The similarity based methods used were BLASTX, BLASTN and BLASTP [54] against the nr and EST databank (NCBI) and BLASTX and TBLASTN against the *A. gambiae *genome and *D. melanogaster *genome using the Ensemble server. Protein domain analysis was performed using SMART and INTERPRO. Stringency parameters were similar to those used in Lobo *et al*. (2003).

### BAC end sequencing

2xYT (2 ml) with 20 ul/ml chloramphenicol was inoculated with 4 ul of precultured BACs and grown for 24 hours with shaking at 37°C. BAC DNA was prepared using the Qiagen R.E.A.L. Prep kit according to the manufacturers instruction. Dye terminator sequencing reactions were set up using 11 ul BAC DNA solution, 2 ul BigDye v3.1, 6 ul 5× buffer, and 7.5 pMol primer (SP6 or T7). Thermal cycling (Applied Biosystems) was carried out at 96°C for 10 min followed by 45 cycles of 96°C for 30 sec, 45°C for 10 sec and 60°C for 4 min. Reactions were precipitated using 80 ul 75% isopropanol, washed using 100 ul 70% ethanol, dried, resuspended in 20 ul HiDi formamide and reactions were analyzed on the ABI Prism^® ^3700 DNA Analyzer.

### Putative Orthology determination

Homology searches were done by submitting Hessian Fly sequences to the BLASTX and TBLASTN program [54] using the PAM30 substitution matrix [55]. An initial list of *Anopheles *and *Drosophila *sequences that had a BLASTX expectation value (E) less than e-4 were selected for manual analysis. This set of sequences was then further verified by direct comparison of the Hessian fly nucleotide and translated sequence to the corresponding *Anopheles *and *Drosophila *entry. *A. gambiae *and *D. melanogaster *sequences were considered to be orthologs of Hessian fly sequences when the E value was less that e-16. [17] or if they satisfied the following criteria: the gene did not belong to a multi-gene family in that particular genome, the TBLASTN value was significant (< e-7) and coincided with the BLASTX sequence chosen for analysis, the E values were significantly higher (> 100×) than that of the next hit (if any) and the *Anopheles *ortholog determined was the previously determined ortholog of the *Drosophila *gene (NCBI, Ensembl). If the gene belonged to a multi-gene family, postulated orthology was based on the degree of significance of the BLASTX and TBLASTN values. In addition, each putative ortholog was searched against its own genome, the top hits selected and phylogenetic trees and molecular evolutionary analyses were conducted (MEGA version 2.1 [56] and ClustalX [57] using all sequences selected for a particular Hessian fly sequence to determine if the sequences clustered as expected. Hessian fly sequences were also analyzed using the BLASTN and BLASTP programs against the available databases.

## Authors' contributions

NFL carried out the sequencing, assembly, gene predictions, putative orthology and synteny studies and drafted the manuscript. SKB and RA performed the genetic mapping, chromosome walking and FISH. MSC carried out the cDNA library construction and sequencing. FHC and JJS initiated and supervised the project, and assisted and approved in the drafting of the final manuscript. All authors read and agreed on the final version of this manuscript.

## Supplementary Material

Additional File 1 Genscan protein predictions of the Mde8i18 BAC clone Click here for file

Additional File 2 Clustal generated multiple sequence alignment of putative protein Mde8i18_5, its Drosophila and Anopheles orthologs, the Human orthologs of these genes as well as the *Anopheles white *gene as an outgroup. Click here for file

## Figures and Tables

**Table 1:** Mean lengths and overlaps between adjacent BAC clones obtained at each step in the chromosome walk Table 1 – Mean lengths and overlaps between adjacent BAC clones obtained at each step in the chromosome walk.

BAC Clones		Mean length ± S.D.(kb)		A-B Overlap
A	B	A	B	Mean ± S.D.(kb)

*Mde 5j15*	*Hf 9c2*	107 ± 6	64 ± 5	35 ± 5
*Hf 9c2*	*Hf 13g20*	64 ± 5	114 ± 9	16 ± 10
*Hf 13g20*	*Mde 15o2*	114 ± 9	86 ± 10	30 ± 14
*Mde 15o2*	*Mde 43n20*	86 ± 10	77 ± 10	29 ± 14
*Mde 43n20*	*Mde 36l15*	77 ± 10	79 ± 10	33 ± 14
*Mde 36l15*	*Hf 15a13*	79 ± 10	115 ± 9	16 ± 15
*Hf 15a13*	*Hf 5l11*	115 ± 9	104 ± 7	70 ± 11
*Hf 5l11*	*Mde 34f2*	104 ± 7	76 ± 4	45 ± 6
*Mde 34f2*	*Mde 47o23*	76 ± 4	93 ± 8	49 ± 11
*Mde 47o23*	*Hf16j4*	93 ± 8	78 ± 6	34 ± 7
*Hf 16j4*	*Hf 15k1*	78 ± 6	88 ± 12	16 ± 10
*Hf 15k1*	*Hf 6k21*	88 ± 12	104 ± 13	50 ± 15

**Table 2:** Relative genetic and physical distances between pairs of STS markers in the contig. The minimal (Min.) and maximal (Max.) physical distances were determined by measuring the distances between the closest and furthest possible limits of each pair of markers

	Genetic distance (cM) between markers	Min.	Max.	Min.	Max.
124–185	2	163	262	81	131
124–233	3	234	339	78	113
124–593	7.5	521	593	69	79
124–291	7.5	549	637	73	85
185–233	1	44	104	44	104
185–593	5.5	331	358	60	65
185–291	5.5	359	402	65	73
233–593	4.5	254	287	79	64
233–291	4.5	282	331	63	73
593-291	0	28	44	-	-
Mean ± SD				68 ± 11	87 ± 22

**Table 3:** *M. destructor *sequences with *A. gambiae *and *D. melanogaster *putative orthologs. The requirements met for the determination of each putative ortholog is below each *Anopheles *or *Drosophila *gene: m) multi-gene family, s) single-gene family, i) BLASTX E value < e^-16^, ii) TBLASTN value < e^-7^, iii) top TBLASTN same as the BLASTX hit, iv) E value was higher (>100×) than that of the next hit in the same gene family, v) the *Anopheles *gene was the predetermined ortholog (NCBI, Ensembl) of the *Drosophila *gene.

***M. destructor***	***A*. ***gambiae*				***D*. ***melanogaster*			
**clone ID**	**Genbank**	**Chromosome location**	**BLASTX E value**	**TBLASTN E value**	**protein ID**	**Chromosome location**	**BLASTX E value**	**TBLASTN E value**

*Mde29L21_SP6*	XM_315112 s,i,ii,iii,v	2L (21A)	1.30E-027	1.70E-013	CG9346 s,i,ii,iii,v	2R (57B)	1.60E-016	1.20E-010
*Mde29L21_T7*	XM_320368 m,i,ii,iii,iv,v	3L (45C)	1.40E-032	1.10E-024	CG1211 m,i,ii,iii,iv,v	3L (61F)	2.20E-008	8.50E-007
*Mde36j2_SP6*	XM_320368 m,i,ii,iii,iv,v	3L (45C)	1.30E-016	6.10E-012	CG1211 m,i,ii,iii,iv,v	3L (61F)	3.10E-008	2.70E-008
*Mde8i18*	XM_320188 m,i,ii,iii,iv,v	3L (46D)	3.10E-036	1.10E-034	CG1609 m,i,ii,iii,iv,v	3R (100B)	2.00E-037	2.50E-032
*Mde8i18/Mde5j15_SP6*	XP_309552 s,i,ii,iii,v	3L (41B)	0.00	0.00	CG2987 s,i,ii,iii,v	2R (60A)	8.40E-218	2.90E-096
*Mde1502_T7*	XM_319149 s,,i,ii,iii	3R (36C)	2.10E-004	8.30E-016	CG13566 s,ii,iii	2R (60A)	5.00E-004	4.40E-009
*Mde33n3_T7*	XM_321918 s,i,ii,iii,v	2R (7B)	1.20E-055	5.50E-045	CG13201 s,i,ii,iii,v	2R (47F)	4.10E-042	5.70E-041
*Hf3a11_T7*	XM_320284 m,i,ii,iii,iv,v	3L (46B)	6.80E-002	1.50E-018	CG18471 s,i,ii,iii,v	2R (53C)	3.70E-012	9.90E-012
*Hf4F24_SP6*	XM_311996 s,i,ii,iii,v	2R (13C)	5.10E-004	6.40E-007	CG1668 m,i,ii,iii,iv,v	X (19DE)	1.50E-004	9.20E-004
*Hf15a13_T7*	XM_310539 s,i,ii,iii,v	X (2B)	2.10E-122	4.00E-123	CG12149 (s,i,ii,iii,v)	X (8E)	7.60E-111	1.80E-115
*Hf10f11_T7*	XM_315277 m,i,ii,iii,iv,v	3L (43B)	7.20E-035	9.20E-033	CG10726 s,i,ii,iii,v	2L (38B	2.00E-024	1.20E-022
